# Blockade of Wnt/*β*-Catenin Pathway Aggravated Silica-Induced Lung Inflammation through Tregs Regulation on Th Immune Responses

**DOI:** 10.1155/2016/6235614

**Published:** 2016-03-16

**Authors:** Wujing Dai, Fangwei Liu, Chao Li, Yiping Lu, Xiaowei Lu, Sitong Du, Ying Chen, Dong Weng, Jie Chen

**Affiliations:** Division of Pneumoconiosis, School of Public Health, China Medical University, No. 77 Puhe Road, Shenyang North New Area, Shenyang 110122, China

## Abstract

CD4^+^ T cells play an important role in regulating silica-induced inflammation and fibrosis. Recent studies showed that Wnt/*β*-catenin pathway could modulate the function and the differentiation of CD4^+^ T cells. Therefore, Wnt/*β*-catenin pathway may participate in the development and progress of silicosis. To investigate the role of Wnt/*β*-catenin pathway, we used lentivirus expressing *β*-catenin shRNA to block the Wnt/*β*-catenin pathway by intratracheal instillation to the mice model of silicosis. Treatment of lentivirus could significantly aggravate the silica-induced lung inflammation and attenuated the fibrosis at the late stage. By analyzing CD4^+^ T cells, we found that blockade of Wnt/*β*-catenin pathway suppressed regulatory T cells (Tregs). Reciprocally, enhanced Th17 response was responsible for the further accumulation of neutrophils and production of proinflammatory cytokines. In addition, blockade of Wnt/*β*-catenin pathway delayed the Th1/Th2 polarization by inhibiting Tregs and Th2 response. These results indicated that Wnt/*β*-catenin pathway could regulate Tregs to modulate Th immune response, which finally altered the pathological character of silicosis. Our study suggested that Wnt/*β*-catenin pathway might be a potential target to treat the silica-induced inflammation and fibrosis.

## 1. Introduction

Silicosis is a damaging kind of pneumoconiosis caused by inhalation of silica mineral dust. The basic pathological changes are diffuse pulmonary fibrosis and silicotic nodule [1, 2]. Inhaled silica particles are firstly recognized by the scavenger receptors on alveolar macrophages and consequently activate alveolar macrophages secreting cytokines and chemokines to induce the influx of inflammatory cells including macrophages, neutrophils, and lymphocytes [3, 4]. Upon the activation of innate immune, silicosis represents acute inflammatory reaction at the early stage, which is characterized by the infiltration of inflammatory cells and destruction of alveolar wall. Accompanied by the emergence of tissue repair, silicosis progresses to diffuse interstitial fibrosis or eventually forms silicotic nodule along with the deposition of extracellular matrix [2]. Adaptive immune is also involved in the development of silicosis. Meanwhile, CD4^+^ T cells are considered as a key participant [5]. Naïve CD4^+^ T cells initiate to differentiate into T helper cells under the activation of TCR signal and the concurrent interaction between costimulatory molecules and specific cytokines, such as Th1, Th2, Th17, and Tregs [6–8]. These distinguishing Th subsets developed complex regulatory networks in response to silica. The progression of silicosis is associated with a disorder of Th1/Th2 balance. Th1 cells characteristically secrete IFN-*γ* to engage in the silica-induced inflammation [9]. Whereas Th2 cells induce fibrogenic response by secreting cytokines such as IL-4 and IL-13, which are known to mediate, fibroblasts differentiate into myofibroblasts [10]. Tregs can modulate this balance to a Th2 dominant response by suppressing Th1 response in a direct or indirect manner [11]. Based on the regulatory role of Tregs in silicosis, Tregs are indicated to possess profibrotic activity. Th17 cells are a recently discovered subset of CD4^+^ T cells which produce IL-17 and are primarily recognized as an important inducer of autoimmune disease [7, 12]. Subsequent studies also reveal the role of Th17 cells in silica-induced lung inflammation and fibrosis [13–17]. The differentiation of Th17 cells depends on the joint action of TGF-*β* and IL-6. Nevertheless, IL-23 is essential to promote the proliferation rather than the generation of Th17 cells [18]. TGF-*β* alone can facilitate the differentiation of Tregs by inducing Foxp3 [19, 20]. However, TGF-*β* plus IL-6 can inhibit Tregs and induce the differentiation of Th17 cells, so there may exist a reciprocal association between Tregs and Th17 cells, and IL-6 may be the regulator of this balance [18, 21].

Canonical Wnt/*β*-catenin is an evolutionarily conserved signaling pathway that controls the embryonic development and cell-fate decision [22, 23]. The amount of *β*-catenin determines the activation of canonical Wnt/*β*-catenin pathway. *β*-catenin is degraded by a destruction complex consisting of axis inhibition protein, adenomatous polyposis coli, casein kinase 1, and glycogen synthase kinase 3*β*. In the presence of Wnt ligands, transmembrane protein Frizzled and its coreceptor LRP5/6 are activated to disrupt the destruction complex; hence *β*-catenin proteins accumulate and then translocate into the nucleus to generate target gene transcription [24]. In the immune system, the role of Wnt/*β*-catenin pathway in thymic T cells development has been fully studied [25]. However, the association between peripheral T lymphocytes differentiation and Wnt/*β*-catenin pathway still needs to be explored. It had been proved that *β*-catenin combined with TCF-1 activated the GATA3 transcription to initiate Th2 differentiation [26, 27]. CD4^+^CD25^+^Tregs expressing stable *β*-catenin inhibited inflammation more effectively due to the superior ability to survive [28]. On the contrary, another study demonstrated that activation of Wnt/*β*-catenin pathway competed with the function of Foxp3 and thus reduced the suppressive ability of Tregs [29]. There are studies that also delineated that inhibition of Wnt/*β*-catenin pathway promoted Th17 differentiation [30, 31]. These suggest that Wnt/*β*-catenin pathway may play an important role in CD4^+^ T cells mediated inflammation or fibrosis.

Our previous study had demonstrated that activation of Wnt/*β*-catenin pathway was involved in the development of experimental silicosis [32]. So we suggested that Wnt/*β*-catenin pathway may participate in regulating silica-induced inflammation and fibrosis. In the current study, we used a lentivirus expressing *β*-catenin shRNA to decrease target mRNA, which continuously blocked the Wnt/*β*-catenin pathway in the lung. We found that blockade of Wnt/*β*-catenin pathway suppressed Tregs and elevated Th17 response. Meanwhile, silica-induced inflammation was significantly aggravated. Blocking Wnt/*β*-catenin pathway also mediated the delay of Th1/Th2 polarization by inhibition of Tregs. This effect impeded the resolution of lung inflammation and restrained the Th2-mediated fibrosis.

## 2. Materials and Methods

### 2.1. Animals

Female C57BL/6 mice were purchased from SLAC Laboratory Animal Co., Ltd. (Shanghai, China), at 6–8 weeks of age. All mice were maintained in specific pathogen-free conditions and fed a standard mouse chow at an environmental temperature of 24 ± 1°C and a 12 h/12 h light/dark cycle with water available ad libitum. The animal study was approved by the Animal Care and Use Committee of China Medical University (CMU62043018), which complies with the National Institutes of Health Guide for the Care and Use of Laboratory Animals.

### 2.2. Reagents

The construction of lentivirus has been described in our previous study [32]. Natural crystalline silica particles (Min-U-Sil 5 ground silica; size distribution: 97% <5 *μ*m diameter, 80% <3 *μ*m diameter; median diameter 1.4 *μ*m) were obtained from the U.S. Silica Company (Frederick, MD). Silica particles were boiled in 1 N HCl to eliminate endotoxins. Prior to exposure, suspensions were autoclaved and then sonicated for 10 min.

### 2.3. Silica Exposure and Experimental Design

All the mice were randomly divided into four groups, as follows: (1) direct oral-tracheal instillation of 0.1 mL sterile saline (saline group); (2) exposure to silica by direct oral-tracheal instillation of 1 mg silica crystals suspended in a total volume of 0.1 mL sterile saline (silica group); (3) direct oral-tracheal instillation of 1 mg silica crystals and 3 × 10^7^ transducing units (TU) of *β*-catenin shRNA suspended in a total volume of 0.1 mL sterile saline (silica + *β*-catenin shRNA group); (4) direct oral-tracheal instillation of 1 mg silica crystals and 3 × 10^7^ transducing units (TU) of NC shRNA suspended in a total volume of 0.1 mL sterile saline (silica + NC shRNA group). The method of exposure of the mice to silica crystals (Min-U-Sil 5) has been previously described [13]. Mice were sacrificed on days 7, 28, and 56 after oral-tracheal instillation.

### 2.4. Bronchoalveolar Lavage and Differential Cell Counts

The lungs of the mice were removed and washed in cold phosphate-buffered saline (PBS) after sacrifice of the mice. Bronchoalveolar lavage fluid (BALF) was obtained by cannulating the trachea and then injecting and retrieving 1 mL aliquots of sterile physiological saline two times. The BALF was centrifuged at 1,500 rpm at 4°C for 8 min. After lysis of red blood cells (RBCs), the BALF cell pellet was washed and resuspended in PBS. Total cell counts were determined using standard hematologic procedures. After cytospin preparation, BALF was stained using the Wright-Giemsa method. Macrophages, neutrophils, and lymphocytes were identified in 200 cells using standard morphologic criteria.

### 2.5. Isolation of Hilar Lymph Nodes

Hilar lymph nodes (HLNs) were harvested, dissected with needles, and digested with 0.25% trypsin for 5 min at 37°C. The digestion reaction was terminated by the addition of 3% fetal bovine serum in PBS, and samples were centrifuged at 1,500 rpm and 4°C for 8 min. The HLN cell pellets were washed and resuspended in PBS.

### 2.6. Flow Cytometry

Cells from the HLNs were resuspended in PBS and stimulated with cell stimulation cocktail (including phorbol-12-myristate-13-acetate (PMA), ionomycin, brefeldin A, and monensin) (2 *μ*L/mL) (eBioscience Inc., San Diego, CA, USA) for 5 h in complete RPMI-1640. Cells were then incubated with anti-mouse CD4 PerCP-Cy5.5 clone: RM4-5 for 20 min at 4°C in the dark. After cellar surface staining, cells were fixed and permeabilized using standard reagents, according to the manufacturer's protocols (eBioscience Inc.). Cells were then stained with an anti-mouse IL-17A PE clone: eBio17B7; an anti-mouse Foxp3 eFluor® 660 clone: FJK-16s; and an anti-mouse IFN-*γ* Alexa Fluor® 488 Clone: XMG1.2 (eBioscience Inc.). Cells were subsequently analyzed using a FACSCanto II flow cytometer (BD Biosciences, Franklin Lakes, NJ). Dead cells and silica particles were gated out according to forward scattering (FSC) and side scattering (SSC). Cells were analyzed with BD FACSDiva*™* Software v6.1.2 (BD Biosciences). Cytokines in BALF were measured by using BD CBA Mouse Th1/Th2/Th17 Cytokine Kit (BD Biosciences, San Jose, CA, USA). The operations were performed according to the manufacturer's instructions. Samples were measured on the FACSCanto II flow cytometer (BD Biosciences, Franklin Lakes, NJ) and analyzed by FCAP Array*™* Software (BD Biosciences, Franklin Lakes, NJ). Individual cytokine concentrations were indicated by their fluorescent intensities. Fluorescence intensity was proportional to the amount of a given cytokine in a vial, and the protein concentrations of the test samples were estimated according to the standard curves acquired after analysis of standard dilutions [33].

### 2.7. RNA Extraction and Real-Time RT-PCR

Total RNA was extracted from lung homogenates using TRIzol reagent (Invitrogen, Carlsbad, CA, USA), according to the manufacturer's protocol. Total lung RNA (2 *μ*g) samples were reverse-transcribed (RT) separately in 20-*μ*L volumes using a program of 37°C for 15 min and 85°C for 5 sec. using ABI 2720 (Applied Biosystems, Foster City, CA, USA). Primers and TaqMan probes sequences were as follows: IL-1*β*, sense 5′-TGACCTGGGCTGTCCTGATG-3′, antisense 5′-GGTGCTCATGTCCTCATCCTG-3′; IL-17A, sense 5′-GCAAAAGTGAGCTCCAGAAGG-3′, antisense 5′-TCTTCATTGCGGTGGAGAGTC-3′; IL-6, sense 5′-CAATTCCAGAAACCGCTATGAAG-3′, antisense 5′-GTAGGGAAGGCCGTGGTTG-3′; IFN-*γ*, sense 5′-AAGCGTCATTGAATCACACCTG-3′, antisense 5′-TGACCTCAAACTTGGCAATACTC-3′; IL-10, sense 5′-GGGGCCAGTACAGCCGGGAA-3′, antisense 5′-CTGGCTGAAGGCAGTCCGCA-3′; Foxp3, sense 5′-AAGCCCCGGAGAGGCAGAGG-3′, antisense 5′-TGCAGGCTCAGGTTGTGGCG-3′; IL-4, sense 5′-AAAATCACTTGAGAGAGATCATCGG-3′; antisense 5′-GTTGCTGTGAGGACGTTTGG-3′; IL-13, sense 5′-CCCCTGTGCAACGGCAGCAT-3′; antisense 5′-GAAGGGGCCGTGGCGAAACA-3′; and GAPDH, sense 5′-CAATGTGTCCGTCGTGGATCT-3′, antisense 5′-GTCCTCAGTGTAGCCCAAGATG-3′. The probe sequences were as follows: IL-1*β*, 5′-(FAM) TCGCAGCAGCACATCAACAAGAGC (BHQ1)-3′; IL-17A, 5′-(FAM) CCTCAGACTACCTCAACCGTTCCAC (BHQ1)-3′; IL-6, 5′-(FAM) CACCAGCATCAGTCCCAAGAAGGCA (BHQ1)-3′; IFN-*γ*, 5′-(FAM) CTTCTTCAGCAACAGCAAGGCGAA (BHQ1)-3′; IL-10, 5′-(FAM) GCACCCACTTCCCAGTCGGCCAGAGCC (BHQ1)-3′; Foxp3, 5′-(FAM) ACCACCCCGCCACCTGGAAGAATGCCA (BHQ1)-3′; IL-4, 5′-(FAM) TGGCGTCCCTTCTCCTGTGACCTCG (BHQ1)-3′; IL-13, 5′-(FAM) TGGACCTGGCCGCTGGCGGGT (BHQ1)-3′; and GAPDH, 5′-(FAM) CGTGCCGCCTGGAGAAACCTGCC (BHQ1)-3′. For T-bet, IL-23, and GATA-3, reverse-transcribed cDNA was detected by SYBR Green on an ABI 7500 system (Applied Biosystems, Carlsbad, CA, USA), according to the manufacturer's protocol. Their primer sequences were as follows: T-bet, sense 5′-TCAACCAGCACCAGACAGAGA-3′, antisense 5′-TCCACCAAGACCACATCCAC-3′; IL-23, sense 5′-ACATGCACCAGCGGGACATA-3′, antisense 5′-CTTTGAAGATGTCAGAGTCAAGCAG-3′; GATA-3, sense 5′-GAAACCGGAAGATGTCTAGCAAA-3′, antisense 5′-TGGAGTGGCTGAAGGGAGA-3′. Real-time RT-polymerase chain reaction (PCR) was performed with a premix Ex Taq RT-PCR kit (DRR039A; Takara, Dalian, China) or a SYBR Premix Ex Taq II RT-PCR kit (DRR081A; Takara). A total of 2 *μ*L of cDNA was used in each 25-*μ*L PCR volume. Each sample was assayed in duplicate. Differences in amplification efficiencies between the target and housekeeping genes were identified by comparing standard curve slopes. Real-time PCR was performed with ABI 7500 (Applied Biosystems) according to the following program: (i) TaqMan: 95°C for 30 sec., 40 cycles of 95°C for 5 sec., and 62°C for 34 sec. or (ii) SYBR Green: 95°C for 30 sec., 40 cycles of 95°C for 5 sec., and 60°C for 34 sec. PCR analyses were performed with ABI 7500 system software.

### 2.8. Pathological Examination

Lung tissues were fixed in 4% paraformaldehyde in PBS. Tissues were embedded in paraffin, cut into 6-*μ*m thick sections, and stained with hematoxylin and eosin (H&E) and Masson's trichrome stain for pathological evaluation of inflammation and fibrosis, respectively. Lung morphology was visualized using an Olympus BX51 microscope at 200x magnification.

### 2.9. Statistics

SPSS 19.0 software was used to conduct statistical analyses. The differences between values were evaluated through one-way analysis of variance (ANOVA) followed by pairwise comparison with the Student-Newman-Keuls test. *p* < 0.05 was considered statistically significant.

## 3. Results

### 3.1. Blockade of Wnt/*β*-Catenin Pathway Aggravated the Silica-Induced Lung Inflammation

**Figure 1:**
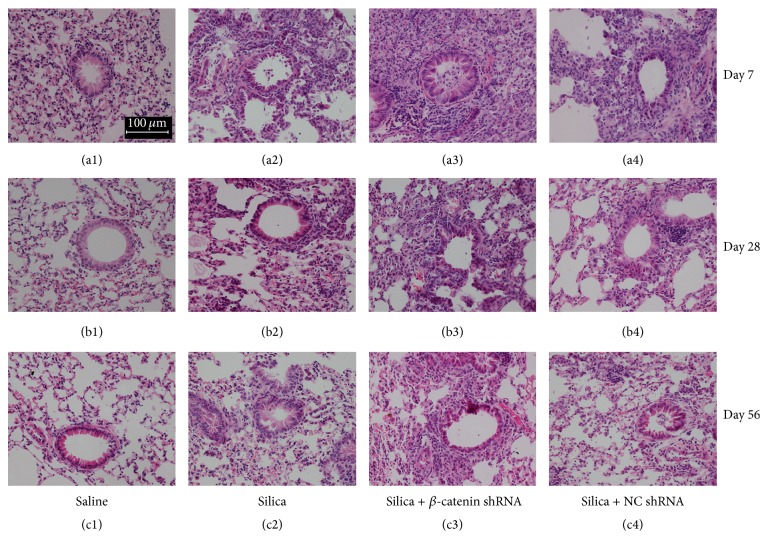
Blockade of Wnt/*β*-catenin pathway aggravated the silica-induced lung inflammation. Lung sections from C57BL/6 were stained with H&E and observed under the light microscope (magnifications ×200). Histopathological changes were assessed in saline group (a1–c1), silica group (a2–c2), silica + *β*-catenin group (a3–c3), and silica + NC shRNA group (a4–c4) at days 7 (a1–a4), 28 (b1–b4), and 56 (c1–c4).

Intratracheal instillation of silica particles could induce acute alveolitis which is characterized by infiltration of inflammatory cells and destruction of alveolar wall structures. To investigate the role of Wnt/*β*-catenin pathway in silica-induced inflammation, we observed the sections of lung tissue stained by H&E. The saline control group showed normal alveolar structure and no infiltration of inflammatory cells at all time points (Figures 1(a1), 1(b1), and 1(c1)). In contrast, silica group and silica + NC shRNA group developed severe pulmonary alveolitis at day 7 due to the treatment of silica. Acute inflammation destructed the intrinsic alveolar structure and accumulated massive inflammatory cells in alveolar septal (Figures 1(a2) and 1(a4)). At day 28, the inflammation attenuated compared with day 7 (Figures 1(b2) and 1(b4)). At day 56, the inflammation basically vanished and the alveolar walls were thickened by tissue repair process (Figures 1(c2) and 1(c4)). However, the silica + *β*-catenin shRNA group exhibited significantly aggravated inflammation compared to silica group and silica + NC shRNA group at day 7 (Figure 1(a3)). At day 28, the exudation and infiltration were alleviated compared with day 7, while the inflammation was apparently severer than the other two groups (Figure 1(b3)). At day 56, the pathological characteristics of silica + *β*-catenin shRNA group were still inflammation (Figure 1(c3)). Thereby, the results of H&E staining showed that blockade of Wnt/*β*-catenin pathway aggravated the silica-induced inflammation at early stage. Besides, the inflammation persisted for longer time compared with silica group and silica + NC shRNA group. Finally, the development of silicosis was delayed by blocking Wnt/*β*-catenin pathway.

### 3.2. Blockade of Wnt/*β*-Catenin Pathway Increased the Accumulation of Inflammatory Cells and the Production of Proinflammatory Cytokines

**Figure 2:**
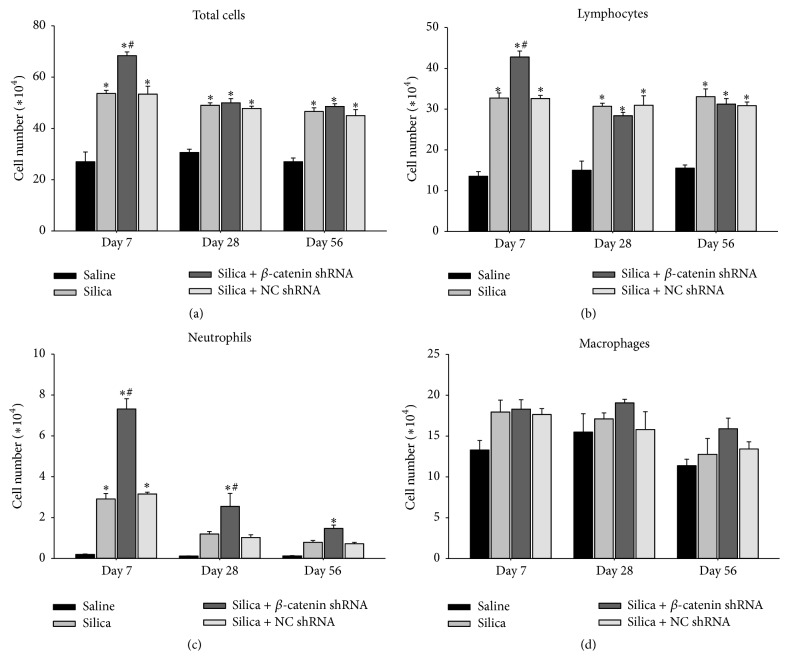
Blockade of Wnt/*β*-catenin pathway increased the accumulation of inflammatory cells. Total cells (a), lymphocytes (b), neutrophils (c), and macrophages (d) in BALF were counted by using Wright-Giemsa staining. Values are shown as mean ± SEM (*n* = 5) (*∗*, as compared with saline group at the same time point, *p* < 0.05; #, as compared with silica group and silica + NC shRNA group at the same time point, *p* < 0.05).

**Figure 3:**
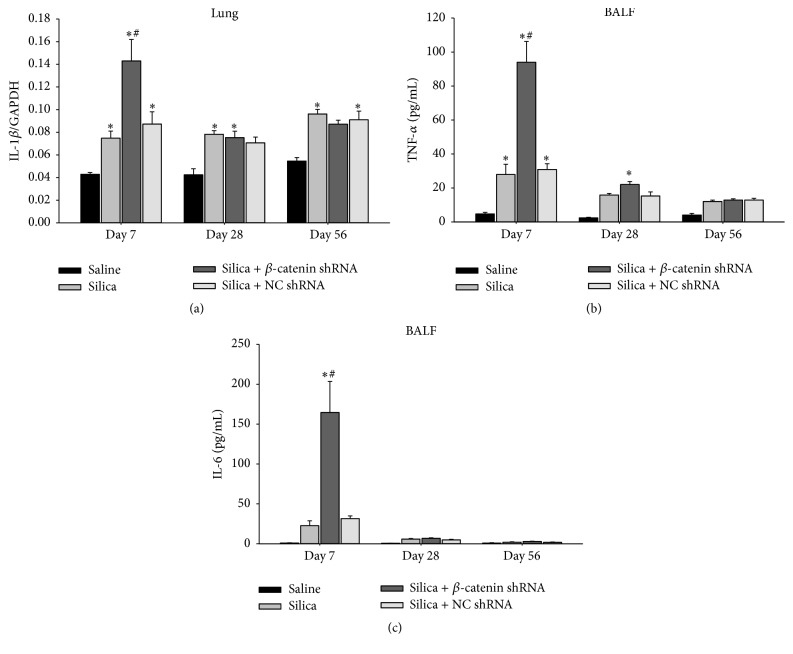
Blockade of Wnt/*β*-catenin pathway increased the production of proinflammatory cytokines. (a) Expression of IL-1*β* mRNA in lung was assayed by real-time RT-PCR using the −ΔΔCt method. The concentrations of TNF-*α* (b) and IL-6 (c) in BALF were assayed by flow cytometry using CBA Kit. Values are shown as mean ± SEM (*n* = 4-5) (*∗*, as compared with saline group at the same time point, *p* < 0.05; #, as compared with silica group and silica + NC shRNA group at the same time point, *p* < 0.05).

In response to silica, alveolar macrophages released abundant cytokines and chemokines. Subsequently, more inflammatory cells were recruited and amplified the inflammation [4]. We analyzed the infiltration of inflammatory cells by counting total cells, lymphocytes, macrophages, and neutrophils in BALF. At three time points, the number of total cells obviously augmented in silica-treated groups compared with saline group. At day 7, more inflammatory cells were accumulated in silica + *β*-catenin shRNA group than other groups (Figure 2(a)). Differential cell counting showed that the number of neutrophils increased obviously at days 7 and 28 in silica + *β*-catenin shRNA group compared with silica group and silica + NC shRNA group (Figure 2(c)). More lymphocytes were also accumulated in silica + *β*-catenin shRNA group than other groups at day 7 (Figure 2(b)), while the number of macrophages showed no difference between three silica-treated groups at all time points (Figure 2(d)). At the early stage of silica-induced inflammation, IL-1*β*, TNF-*α*, and IL-6 exerted important proinflammatory function. Our results showed that the expression of IL-1*β* mRNA in lung and the concentration of TNF-*α* and IL-6 cytokines in BALF increased obviously in silica + *β*-catenin shRNA group at day 7 compared with silica group and silica + NC shRNA group. Afterwards, these cytokines dramatically declined to a low level in silica-treated groups at days 28 and 56 (Figures 3(a)–3(c)). Above all, our study revealed that blockade of Wnt/*β*-catenin pathway aggravated silica-induced inflammation by increasing the accumulation of inflammatory cells and the production of proinflammatory cytokines.

### 3.3. Th17 Response Was Enhanced by Blocking the Wnt/*β*-Catenin Pathway

**Figure 4:**
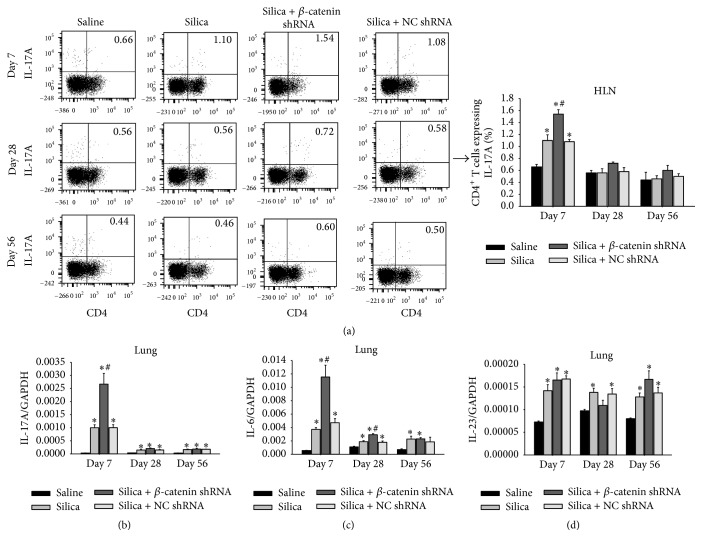
Th17 response was enhanced by blocking the Wnt/*β*-catenin pathway. (a) The percentage of IL-17A^+^CD4^+^ T cells in HLN was calculated by flow cytometry; data were shown as scatter plot and graph. Expressions of IL-17A (b), IL-6 (c), and IL-23 (d) in lung were assayed by real-time RT-PCR using the −ΔΔCt method. Values are shown as mean ± SEM (*n* = 4-5) (*∗*, as compared with saline group at the same time point, *p* < 0.05; #, as compared with silica group and silica + NC shRNA group at the same time point, *p* < 0.05).

It has been well established that CD4^+^ T cells played an important role in silicosis. However, recent studies found that Wnt/*β*-catenin pathway was involved in the development of CD4^+^ T cells [27, 28]. To explore the potential mechanism that blockade of Wnt/*β*-catenin pathway aggravated silica-induced inflammation, we investigated Th response by analyzing different subsets of Th cells. Th17 cells functioned to induce inflammation and autoimmune diseases by producing IL-17A [34]. Instillation of silica particles apparently activated the differentiation of Th17 cells and the secretion of IL-17A at the early stage of silicosis. In this study, we evaluated the Th17 response by flow cytometry and real-time RT-PCR. The percentage of CD4^+^IL-17A^+^ T cells in HLN was higher in silica + *β*-catenin shRNA group at day 7 (Figure 4(a)). Compared with silica group and silica + NC shRNA group, expressions of IL-17A, IL-21, and ROR*γ*t mRNA significantly increased in silica + *β*-catenin shRNA group at day 7 (Figure 4(b) and Figure S1 in Supplementary Material available online at http://dx.doi.org/10.1155/2016/6235614). We then analyzed cytokines which were crucial for the development of Th17 cells. The level of IL-6 mRNA in lung tissue significantly increased in silica + *β*-catenin shRNA group at days 7 and 28 (Figure 4(c)). But IL-23 mRNA showed no difference at three time points (Figure 4(d)). These results indicated that blockade of Wnt/*β*-catenin pathway could enhance the Th17 response.

### 3.4. Th1 Response Was Enhanced by Blocking the Wnt/*β*-Catenin Pathway

**Figure 5:**
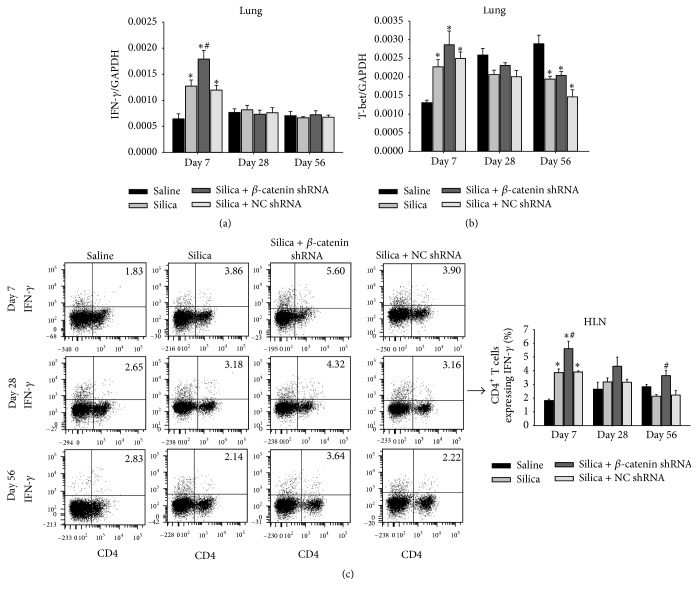
Th1 response was enhanced by blocking the Wnt/*β*-catenin pathway. Expressions of IFN-*γ* (a) and T-bet (b) in lung were assayed by real-time RT-PCR using the −ΔΔCt method. (c) The percentage of IFN-*γ* ^+^CD4^+^ T cells in HLN was calculated by flow cytometry; data were shown as scatter plot and graph. Values are shown as mean ± SEM (*n* = 4-5) (*∗*, as compared with saline group at the same time point, *p* < 0.05; #, as compared with silica group and silica + NC shRNA group at the same time point, *p* < 0.05).

Th1 cells and Th1-related cytokines were increased at the early stage of silicosis. This suggested an important role of Th1 response in governing the inflammation of silicosis [9]. We assayed the mRNA of IFN-*γ* by real-time RT-PCR. At day 7, the expression of IFN-*γ* mRNA was higher in silica + *β*-catenin shRNA group compared with silica group and silica + NC shRNA group. Followed by the progress of silicosis, IFN-*γ* mRNA decreased to an equivalent level in silica-treated groups at days 28 and 56 (Figure 5(a)). T-bet is Th1-specific transcription factor. Activation of T-bet initiates the differentiation of Th1 subset and promotes the secretion of IFN-*γ* [35]. The level of T-bet mRNA showed a similar tendency in comparison to IFN-*γ* (Figure 5(b)). Then we examined Th1 cells in HLN by flow cytometry. The percentage of CD4^+^IFN-*γ* ^+^ T cells in silica + *β*-catenin shRNA group increased at day 7 compared with silica group and silica + NC shRNA group (Figure 5(c)). Overall, Th1 response was elevated due to the blockade of Wnt/*β*-catenin pathway during the silica-induced lung inflammatory stage.

### 3.5. Blockade of Wnt/*β*-Catenin Pathway Impaired Tregs

**Figure 6:**
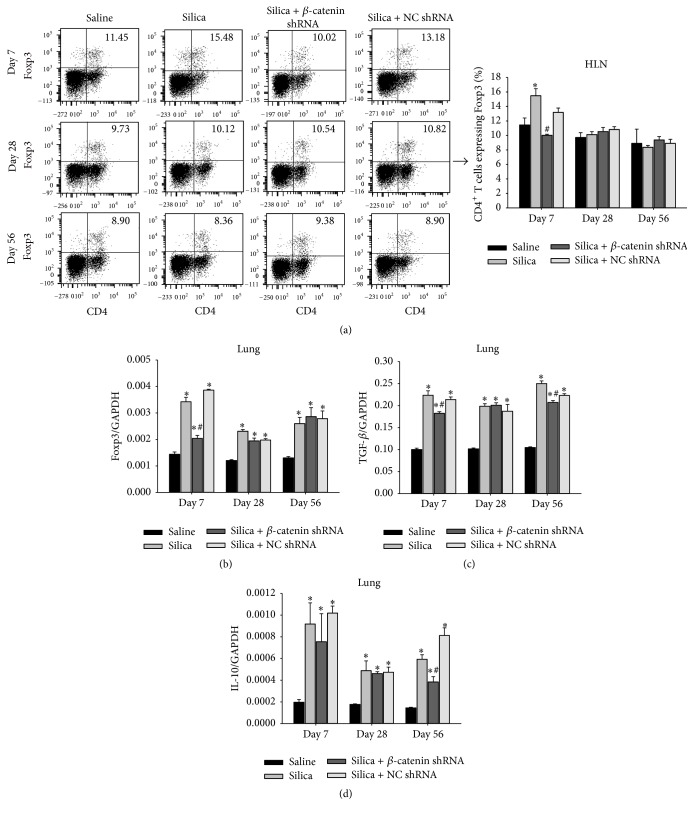
Blockade of Wnt/*β*-catenin pathway impaired Tregs. (a) The percentage of Foxp3^+^CD4^+^ T cells in HLN was calculated by flow cytometry; data were shown as scatter plot and graph. Expressions of Foxp3 (b), TGF-*β* (c), and IL-10 (d) in lung were assayed by real-time RT-PCR using the −ΔΔCt method. Values are shown as mean ± SEM (*n* = 3–5) (*∗*, as compared with saline group at the same time point, *p* < 0.05; #, as compared with silica group and silica + NC shRNA group at the same time point, *p* < 0.05).

Tregs are a distinct subset of CD4^+^ T cells which maintain the immune homeostasis and exert self-tolerance [36, 37]. Decreased amount or function of Tregs resulted in severe lung inflammation in experimental silicosis [11]. We used flow cytometry to determine the percentage of CD4^+^Foxp3^+^ T cells in HLN. At day 7, Tregs decreased compared with silica group and silica + NC shRNA group. There was no difference between all groups at days 28 and 56 (Figure 6(a)). Foxp3 is the specific transcription factor of Tregs. In accordance with Tregs, Foxp3 decreased obviously at day 7 in silica + *β*-catenin shRNA group. The reduction of Foxp3 became indistinctive among silica-treated groups at days 28 and 56 (Figure 6(b)).* In vivo*, Tregs negatively regulate other cell types by producing cytokines such as TGF-*β* and IL-10. We then examined the expression of TGF-*β* and IL-10 mRNA in lung tissue to assess the suppressive capacity of Tregs. TGF-*β* reduced at day 7 and day 56 compared with silica group and silica + NC shRNA group. Similar to TGF-*β*, IL-10 decreased at day 7 (no significance) and day 56 in silica + *β*-catenin shRNA group (Figures 6(c)-6(d)). These data suggested that blockade of Wnt/*β*-catenin pathway inhibited Tregs and impaired the Treg-mediated suppression.

### 3.6. Blockade of Wnt/*β*-Catenin Pathway Alleviated Silica-Induced Fibrosis

**Figure 7:**
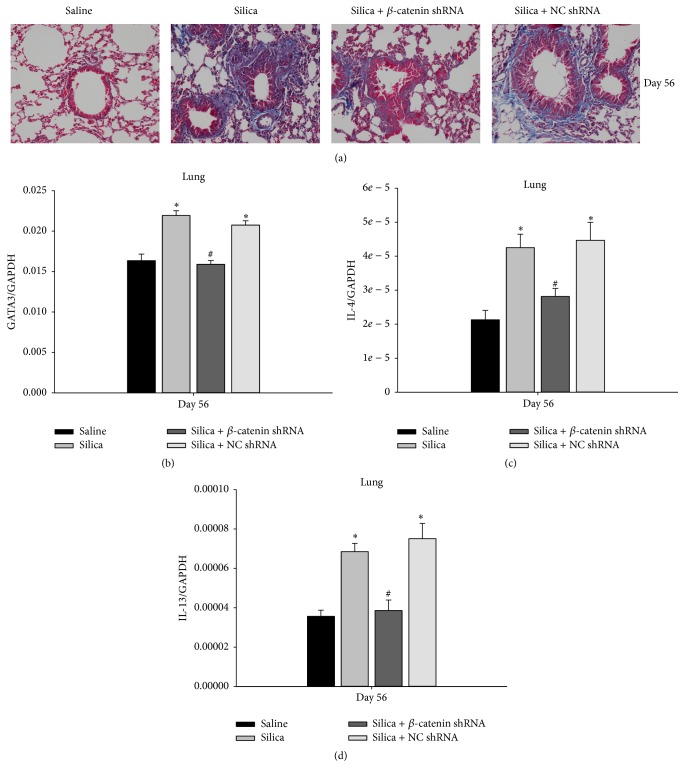
Blockade of Wnt/*β*-catenin pathway alleviated silica-induced fibrosis. (a) Lung sections from C57BL/6 were stained with Masson trichrome staining and observed under the light microscope (magnifications ×200). Expressions of GATA3 (b) and IL-4 and IL-13 (c) in the lung were assayed by real-time RT-PCR using the −ΔΔCt method. Values are shown as mean ± SEM (*n* = 4-5) (*∗*, as compared with saline group, *p* < 0.05; #, as compared with silica group and silica + NC shRNA group, *p* < 0.05).

Followed by the degradation of inflammation, collagen deposited in the alveolar septum and gradually formed interstitial fibrosis. In order to investigate the level of fibrosis, Masson's trichrome blue staining was applied to colorize the collagen in paraffin sections of lung tissue. At the late stage of experimental silicosis, there was no collagen deposition in saline group. The accumulation of collagen was obvious in silica group and silica + NC shRNA group. However, silica + *β*-catenin shRNA group showed significantly less collagen deposition (Figure 7(a)). Th1/Th2 polarization drives the progress of experimental silicosis that develops from an inflammatory stage to fibrosis stage. In this course, Th2 response supersedes Th1 response and generates cytokines to promote the production of collagen. A study has proved that deletion of TCF-1, which was a downstream transcription factor of Wnt/*β*-catenin pathway, impaired the Th2 response by affecting the activation of GATA3 [27]. Our results showed a similar phenomenon that GATA3 mRNA was decreased in silica + *β*-catenin shRNA group at day 56 (Figure 7(b)). Moreover, profibrogenic cytokines secreted by Th2 cells were further examined in this study; the level of IL-4 and IL-13 mRNA at day 56 was lower compared with silica group and silica + NC shRNA group (Figures 7(c) and 7(d)). These data demonstrated that using *β*-catenin shRNA to block Wnt/*β*-catenin pathway could attenuate silica-induced fibrosis by suppressing Th2 response.

## 4. Discussion

**Figure 8:**
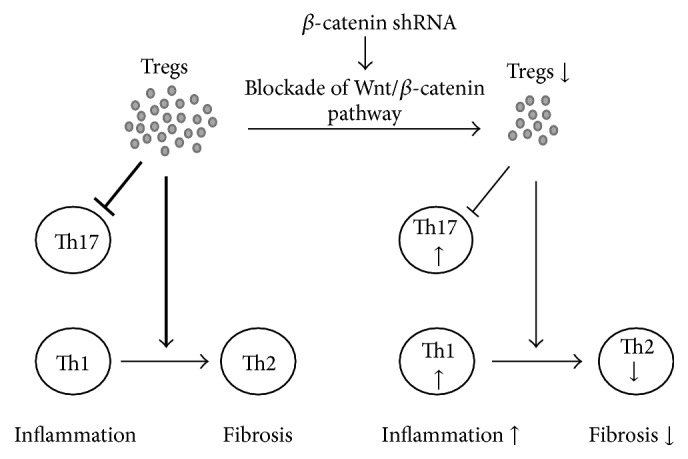
A schematic representation for the modulation of Th immune responses by blocking Wnt/*β*-catenin pathway in silicosis.

Silicosis is a kind of serious occupational lung disease. It is still incurable nowadays. Inhaled silica particles firstly induce the accumulation of inflammatory cells and cause lung inflammation. Followed by the inflammation, adaptive immunity drives the pathological progress of silicosis entering a fibrotic phase [38]. The outcome of silicosis is diffuse pulmonary interstitial fibrosis or even silicotic nodule, which is gravely destructive for the patient's respiratory function. So exploring the mechanism of silicosis is of profound significance to provide effective therapeutic methods for patients. Many studies have proved that CD4^+^ T lymphocytes participated in the pathogenesis of silicosis [39–42]. Our previous studies also delineated that different subsets of CD4^+^ T cells regulated the pathology of silicosis arranging from inflammation stage to fibrosis stage [11, 14–17]. Wnt signaling is an evolutionary conserved pathway. Extensive evidences have demonstrated the close connection between T lymphocytes and Wnt/*β*-catenin pathway. However, researches mainly focused on the role of Wnt/*β*-catenin pathway in the T cell development in thymus. The functions of Wnt/*β*-catenin pathway on peripheral T cell differentiation are still not well understood. In this study, we explored the change of Th immune response by blocking Wnt/*β*-catenin pathway in an experimental silicosis model. Combined with the pathological examination, we revealed the regulatory role of Wnt/*β*-catenin pathway in silicosis. Our study demonstrated that the severity of silica-induced lung inflammation was significantly enhanced by blocking Wnt/*β*-catenin pathway. However, the fibrotic response was ameliorated at the late stage. Furthermore, the mechanism was elucidated by investigating the regulatory role of Wnt/*β*-catenin pathway on CD4^+^ T cells (Figure 8). We suggested that Wnt/*β*-catenin pathway might act on CD4^+^ T cells to regulate the silica-induced inflammation and fibrosis.

As a crucial protein in the canonical Wnt pathway, the amount of *β*-catenin in cytoplasm determines whether *β*-catenin could translocate into the nucleus, bind to TCF/LEF, and activate target genes. We used a lentivirus expressing shRNA to specifically degrade *β*-catenin mRNA. Consequently, the Wnt/*β*-catenin pathway was continuously blocked by this method. Our previous study has verified the efficiency of the lentivirus in both* in vitro* and* in vivo* experiments [32]. In this study, lentivirus was administered through intratracheal injection to block the Wnt/*β*-catenin pathway in the lung. By observing H&E staining sections of lung tissues, we found that treatment of silica + *β*-catenin shRNA induced remarkable severer inflammation than other groups at all time points. This indicated that blockade of Wnt/*β*-catenin pathway in the lung exacerbated the silica-induced inflammation. In addition, the progress of experimental silicosis was also delayed due to the continuous inflammatory response. Paralleled with the results of H&E staining, blockade of Wnt/*β*-catenin pathway showed extensive accumulation of inflammatory cells in the lung. Among them, neutrophils and lymphocytes were the major cell types that increased significantly. Proinflammatory cytokines TNF-*α*, IL-6, and IL-1*β* were also abundantly secreted at the early stage in silica + *β*-catenin shRNA group. These suggested that blocking the Wnt/*β*-catenin pathway in lung promoted the accumulation of inflammatory cells and secretion of proinflammatory cytokines, which ultimately led to an amplified silica-induced lung inflammation.

Although innate immune processes are proved to be sufficient for driving silicosis, Th responses are still thought to play an important regulatory role in the pathogenesis of silicosis [43]. Th17 response has been proved to participate in the lung inflammation during experimental silicosis. The level of Th17 cells and IL-17A in lung tissue obviously elevated after treating mice with silica particles. Accordingly, IL-17R^−/−^ mice or mice injected with anti-IL-17A neutralizing Abs showed significantly limited inflammatory response. This indicated that Th17 cells possessed an obvious proinflammatory role in silica-induced inflammation [13–15, 44]. The proinflammatory properties of Th17 cells are mainly implemented by IL-17A. IL-17RA is the receptor for IL-17A. As IL-17RA is distributed in multiple tissues, IL-17A is effective in promoting inflammation by acting on various cell types to induce the production of cytokines and chemokines such as IL-6, TNF-*α*, IL-1*β*, CXCL1, and CXCL8 [45]. Blockade of Wnt/*β*-catenin pathway significantly enhanced Th17 response at the early stage of silicosis. As a result, the increased IL-17A promoted the infiltration of inflammatory cells, augmented the production of proinflammatory cytokines, and induced severe lung inflammation. To investigate the mechanism of Th17 enhancement, we analyzed the expression of IL-6 and IL-23, which were critical for the commitment of Th17 lineage. IL-6 is a pleiotropic cytokine mainly derived from innate immune system such as macrophages and DCs [46]. IL-6 combined with TGF-*β* can persistently activate STAT3 signaling to initiate the differentiation of Th17 cells by inducing the transcription of ROR*γ*t [47, 48]. IL-23 was not essential for the differentiation of Th17 cells, but it acted to sustain the proliferation of Th17 cells [49]. Our study showed that blockade of Wnt/*β*-catenin pathway significantly increased IL-6 rather than IL-23 at the early stage. This resulted from the enhanced Th17 response, and IL-6 in turn promoted the differentiation of Th17 cells. The mutual stimulation of IL-6 and Th17 cells amplified the silica-induced inflammation by blocking Wnt/*β*-catenin pathway. What is more, Th1 response was also boosted by blocking Wnt/*β*-catenin pathway. Considering the fact that Th1 cells produce IFN-*γ* to active macrophages and sustain the infiltration of macrophages, these suggested that the aggravating inflammation was regulated by the enhanced Th17/Th1 response.

Tregs are an important T cell lineage which function to maintain the immune homeostasis and self-tolerance [36]. In experimental silicosis, Tregs suppress the silica-induced inflammation by negatively regulating the immune response. Deficiency of Tregs would induce an intense inflammatory response [11]. The development of Tregs is orchestrated by a series of signaling pathways. Recent studies have focused on Wnt/*β*-catenin pathway and demonstrated its participation in regulating Tregs. One study that constructed Tregs expressing stable form of *β*-catenin proved that activation of Wnt/*β*-catenin pathway enhanced the survival of Tregs* in vitro* [28]. However, another research found that the suppression mediated by Tregs was diminished by the activation of Wnt/*β*-catenin pathway [29]. Thus, the role of this signaling pathway on Tregs is still controversial. In our study, Tregs were reduced in HLN at the early stage by blocking Wnt/*β*-catenin pathway. Paralleled with the change of Tregs, expression of Foxp3 mRNA in lung also decreased. Tregs could secrete cytokines such as TGF-*β* and IL-10 to suppress the immune response [50]. The level of TGF-*β* and IL-10 mRNA also decreased at the early stage. Taken together, we suggested that Tregs were impaired by blocking Wnt/*β*-catenin pathway. Although Wnt/*β*-catenin signaling pathway was demonstrated to negatively modulate the function of Tregs [29], blockade of Wnt/*β*-catenin should enhance the suppressive ability of Tregs. However, exposure to silica induced the apoptosis of CD4^+^ T cells [51]; gene silencing of *β*-catenin during silicosis may extinguish the antiapoptotic property contributed by Wnt pathway. As a result, the survival of Tregs was weakened and the amount of Tregs was reduced, which might outcompete the enhanced function of Tregs. It was explicit that there existed a reciprocal relationship between Tregs and Th17 cells. Inhibition of Tregs by blocking Wnt/*β*-catenin pathway might promote the Th17 response and induce an aggravating silica-induced inflammation.

The pathogenesis of experimental silicosis contained a shift from Th1 response to Th2 response and this polarization was regulated by Tregs. The Th1/Th2 balance determined the progress of pulmonary fibrosis induced by silica. Delaying the Th1/Th2 polarization might slow down the resolution of lung inflammation, and Th2 response mediated lung fibrosis would be postponed. On one hand, our study showed that Th1 response was enhanced by the treatment of *β*-catenin shRNA at the early stage and was still relatively higher at the late stage. On the other hand, we found that blockade of Wnt/*β*-catenin pathway inhibited GATA-3, which was the specific nuclear transcription factor of Th2 cells, as well as reducing the profibrogenic cytokines IL-4 and IL-13. These results were consistent with the evidence that *β*-catenin binding to TCF-1 promoted Th2 response and inhibited Th1 response by initiating GATA-3 expression [27]. So blocking the Wnt/*β*-catenin pathway delayed the Th1/Th2 polarization by impairing Tregs and by directly inhibiting the differentiation of Th2 cells, based on the notion that Th2 response was distinctly profibrotic. The morphological results were consistent with the tendency of Th1/Th2 polarization, which was exhibited as the attenuated fibrosis at the late stage and the seriously persistent inflammation. Except for Th2 cells, it is not surprising that Tregs may possess profibrotic properties for the highly secreted TGF-*β*, which is identified as a critical fibrogenic mediator and is also a potent anti-inflammatory cytokine [52, 53]. However, the concept of whether Tregs can protect or worsen tissues from fibrosis is still controversial. The observation that knockout of mdig ameliorates silica-induced lung fibrosis by altering the Th17/Tregs balance supported Tregs to be antifibrogenic [54]. However, some other studies demonstrated that Tregs exacerbated the silica-induced fibrosis via direct (producing PDGF-B and TGF-*β*) or indirect (modulating Th1/Th2 polarization) mechanisms [11, 41]. In this study, inhibition of Tregs and Th2 response by blocking Wnt/*β*-catenin pathway could attenuate silica-induced fibrosis. This suggested that the Tregs/Th2 axis might play profibrotic activities.

## 5. Conclusions

Our study demonstrated that blockade of Wnt/*β*-catenin pathway could inhibit Tregs and enhance Th17 response reciprocally. This effect promoted the production of massive proinflammatory cytokines and led to an aggravating silica-induced inflammation. On the other hand, blocking Wnt/*β*-catenin pathway delayed the Th1/Th2 polarization by inhibiting Tregs and Th2 response. As a result, the inflammation was sustained whereas the fibrosis attenuated at the late stage of experimental silicosis.

## Supplementary Material

Figure S1: Th17 response was enhanced by blocking the Wnt/*β*-catenin pathway. Expression of IL-21 (A) and ROR*γ*t (B) in lung were assayed by realtime RT-PCR using the −ΔΔCt method. Values are shown as mean ± SEM (*n*=4-5). (∗, as compared with saline group at the same time point, *p*<0.05; #, as compared with silica group and silica+NC shRNA group at the same time point, *p*<0.05).

## Abbreviations

shRNA: Short hairpin RNA
BALF: Bronchoalveolar lavage fluid
HLN: Hilar lymph node
GAPDH: Glyceraldehyde-3-phosphate dehydrogenase
IL-17A: Interleukin-17A
IL-6: Interleukin-6
IL-23: Interleukin-23
IL-1*β*: Interleukin-1 beta
IL-4: Interleukin-4
IL-13: Interleukin-13
IL-10: Interleukin-10
TNF-*α*: Tumor necrosis factor-alpha
IFN-*γ*: Interferon-gamma
Tregs: Regulatory T cells
Th1: T helper type 1 cells
Th2: T helper type 2 cells
Th17: T helper type 17 cells
T-bet: T-cell-specific T-box transcription factor
GATA-3: GATA binding protein-3
Foxp3: Forkhead box P3
ROR-*γ*t: RAR-related orphan receptor-gamma
TGF-*β*: Transforming growth factor-beta
CD4: Cluster of Differentiation 4 receptors.

## References

[1] Gibbs A. R., Wagner J. C., Churg A., Green F. H. Y. (1988). Diseases due to silica. *Pathology of Occupational Lung Disease*.

[2] Weill H., Jones R., Parkes W. (1994). Silicosis and related diseases. *Occupational Lung Disorders*.

[3] Thakur S. A., Hamilton R., Pikkarainen T., Holian A. (2009). Differential binding of inorganic particles to MARCO. *Toxicological Sciences*.

[4] Mossman B. T., Churg A. (1998). Mechanisms in the pathogenesis of asbestosis and silicosis. *American Journal of Respiratory and Critical Care Medicine*.

[5] Suzuki N., Ohta K., Horiuchi T. (1996). T lymphocytes and silica-induced pulmonary inflammation and fibrosis in mice. *Thorax*.

[6] Mosmann T. R., Coffman R. L. (1989). TH1 and TH2 cells: different patterns of lymphokine secretion lead to different functional properties. *Annual Review of Immunology*.

[7] Harrington L. E., Hatton R. D., Mangan P. R. (2005). Interleukin 17-producing CD4^+^ effector T cells develop via a lineage distinct from the T helper type 1 and 2 lineages. *Nature Immunology*.

[8] Josefowicz S. Z., Lu L.-F., Rudensky A. Y. (2012). Regulatory T cells: mechanisms of differentiation and function. *Annual Review of Immunology*.

[9] Garn H., Friedetzky A., Kirchner A., Jäger R., Gemsa D. (2000). Experimental silicosis: a shift to a preferential IFN-*γ*-based Th1 response in thoracic lymph nodes. *The American Journal of Physiology—Lung Cellular and Molecular Physiology*.

[10] Barbarin V., Arras M., Misson P. (2004). Characterization of the effect of interleukin-10 on silica-induced lung fibrosis in mice. *American Journal of Respiratory Cell and Molecular Biology*.

[11] Liu F., Liu J., Weng D. (2010). CD4+CD25+Foxp3+ regulatory T cells depletion may attenuate the development of silica-induced lung fibrosis in mice. *PLoS ONE*.

[12] Park H., Li Z., Yang X. O. (2005). A distinct lineage of CD4 T cells regulates tissue inflammation by producing interleukin 17. *Nature Immunology*.

[13] Lo Re S., Dumoutier L., Couillin I. (2010). IL-17A-producing *γδ* T and Th17 lymphocytes mediate lung inflammation but not fibrosis in experimental silicosis. *The Journal of Immunology*.

[14] Chen Y., Li C., Weng D. (2014). Neutralization of interleukin-17A delays progression of silica-induced lung inflammation and fibrosis in C57BL/6 mice. *Toxicology and Applied Pharmacology*.

[15] Song L., Weng D., Dai W. (2015). Th17 can regulate silica-induced lung inflammation through an IL-1*β*-dependent mechanism. *Journal of Cellular and Molecular Medicine*.

[16] Tang W., Liu F., Chen Y. (2014). Reduction of IL-17A might suppress the Th1 response and promote the Th2 response by boosting the function of treg cells during silica-induced inflammatory response in vitro. *Mediators of Inflammation*.

[17] Song L., Weng D., Liu F. (2012). Tregs promote the differentiation of Th17 cells in silica-induced lung fibrosis in mice. *PLoS ONE*.

[18] Bettelli E., Carrier Y., Gao W. (2006). Reciprocal developmental pathways for the generation of pathogenic effector TH17 and regulatory T cells. *Nature*.

[19] Kretschmer K., Apostolou I., Hawiger D., Khazaie K., Nussenzweig M. C., von Boehmer H. (2005). Inducing and expanding regulatory T cell populations by foreign antigen. *Nature Immunology*.

[20] Chen W., Jin W., Hardegen N. (2003). Conversion of peripheral CD4^+^ CD25^−^ naive T cells to CD4^+^ CD25^+^ regulatory T cells by TGF-*β* induction of transcription factor *Foxp3*. *The Journal Of Experimental Medicine*.

[21] Korn T., Bettelli E., Oukka M., Kuchroo V. K. (2009). IL-17 and Th17 cells. *Annual Review of Immunology*.

[22] Clevers H. (2006). Wnt/*β*-catenin signaling in development and disease. *Cell*.

[23] Reya T., Clevers H. (2005). Wnt signalling in stem cells and cancer. *Nature*.

[24] Staal F. J. T., van Noort M., Strous G. J., Clevers H. C. (2002). Wnt signals are transmitted through N-terminally dephosphorylated *β*-catenin. *EMBO Reports*.

[25] Staal F. J. T., Luis T. C., Tiemessen M. M. (2008). WNT signalling in the immune system: WNT is spreading its wings. *Nature Reviews Immunology*.

[26] Notani D., Gottimukkala K. P., Jayani R. S. (2010). Global regulator SATB1 recruits *β*-catenin and regulates T_H_2 differentiation in Wnt-dependent manner. *PLoS Biology*.

[27] Yu Q., Sharma A., Oh S. Y. (2009). T cell factor 1 initiates the T helper type 2 fate by inducing the transcription factor GATA-3 and repressing interferon-*γ*. *Nature Immunology*.

[28] Ding Y., Shen S., Lino A. C., Curotto de Lafaille M. A., Lafaille J. J. (2008). Beta-catenin stabilization extends regulatory T cell survival and induces anergy in nonregulatory T cells. *Nature Medicine*.

[29] van Loosdregt J., Fleskens V., Tiemessen M. M. (2013). Canonical Wnt signaling negatively modulates regulatory T cell function. *Immunity*.

[30] Lee Y.-S., Lee K.-A., Yoon H.-B. (2012). The Wnt inhibitor secreted Frizzled-Related Protein 1 (sFRP1) promotes human Th17 differentiation. *European Journal of Immunology*.

[31] Suryawanshi A., Manoharan I., Hong Y. (2015). Canonical Wnt signaling in dendritic cells regulates Th1/Th17 responses and suppresses autoimmune neuroinflammation. *The Journal of Immunology*.

[32] Wang X., Dai W., Wang Y., Gu Q., Yang D., Zhang M. (2015). Blocking the Wnt/*β*-catenin pathway by lentivirus-mediated short hairpin RNA targeting *β*-catenin gene suppresses silica-induced lung fibrosis in mice. *International Journal of Environmental Research and Public Health*.

[33] Szaryńska M., Myśliwski A., Myśliwska J., Kmieć Z., Preis K., Zabul P. (2015). Cytokine profiles during delivery affect cord blood hematopoietic stem and progenitors cells. *Cellular Immunology*.

[34] Kolls J. K., Lindén A. (2004). Interleukin-17 family members and inflammation. *Immunity*.

[35] Szabo S. J., Kim S. T., Costa G. L., Zhang X., Fathman C. G., Glimcher L. H. (2000). A novel transcription factor, T-bet, directs Th1 lineage commitment. *Cell*.

[36] Sakaguchi S., Ono M., Setoguchi R. (2006). Foxp3^+^CD25^+^CD4^+^ natural regulatory T cells in dominant self-tolerance and autoimmune disease. *Immunological Reviews*.

[37] Wing K., Sakaguchi S. (2010). Regulatory T cells exert checks and balances on self tolerance and autoimmunity. *Nature Immunology*.

[38] Rimal B., Greenberg A. K., Rom W. N. (2005). Basic pathogenetic mechanisms in silicosis: current understanding. *Current Opinion in Pulmonary Medicine*.

[39] Tripathi S. S., Mishra V., Shukla M. (2010). IL-6 receptor-mediated lung Th2 cytokine networking in silica-induced pulmonary Wbrosis. *Archives of Toxicology*.

[40] Arras M., Huaux F., Vink A. (2001). Interleukin-9 reduces lung fibrosis and type 2 immune polarization induced by silica particles in a murine model. *American Journal of Respiratory Cell and Molecular Biology*.

[41] Lo Re S., Lecocq M., Uwambayinema F. (2011). Platelet-derived growth factor-producing CD4^+^ Foxp3^+^ regulatory T lymphocytes promote lung fibrosis. *American Journal of Respiratory and Critical Care Medicine*.

[42] Barbarin V., Xing Z., Delos M., Lison D., Huaux F. (2005). Pulmonary overexpression of IL-10 augments lung fibrosis and Th2 responses induced by silica particles. *The American Journal of Physiology—Lung Cellular and Molecular Physiology*.

[43] Beamer C. A., Migliaccio C. T., Jessop F., Trapkus M., Yuan D., Holian A. (2010). Innate immune processes are sufficient for driving silicosis in mice. *Journal of Leukocyte Biology*.

[44] Ouyang W., Kolls J. K., Zheng Y. (2008). The biological functions of T Helper 17 cell effector cytokines in inflammation. *Immunity*.

[45] Moseley T. A., Haudenschild D. R., Rose L., Reddi A. H. (2003). Interleukin-17 family and IL-17 receptors. *Cytokine & Growth Factor Reviews*.

[46] Van Snick J. (1990). Interleukin-6: an overview. *Annual Review of Immunology*.

[47] Yang X. O., Pappu B. P., Nurieva R. (2008). T helper 17 lineage differentiation is programmed by orphan nuclear receptors ROR*α* and ROR*γ*. *Immunity*.

[48] Yang X. O., Panopoulos A. D., Nurieva R. (2007). STAT3 regulates cytokine-mediated generation of inflammatory helper T cells. *The Journal of Biological Chemistry*.

[49] Iwakura Y., Ishigame H. (2006). The IL-23/IL-17 axis in inflammation. *Journal of Clinical Investigation*.

[50] Josefowicz S. Z., Rudensky A. (2009). Control of regulatory T cell lineage commitment and maintenance. *Immunity*.

[51] Borges V. M., Lopes M. F., Falcão H. (2002). Apoptosis underlies immunopathogenic mechanisms in acute silicosis. *American Journal of Respiratory Cell and Molecular Biology*.

[52] Shull M. M., Ormsby I., Kier A. B. (1992). Targeted disruption of the mouse transforming growth factor-*β*1 gene results in multifocal inflammatory disease. *Nature*.

[53] Border W. A., Noble N. A. (1994). Transforming growth factor *β* in tissue fibrosis. *The New England Journal of Medicine*.

[54] Thakur C., Wolfarth M., Sun J. (2015). Oncoprotein mdig contributes to silica-induced pulmonary fibrosis by altering balance between Th17 and Treg T cells. *Oncotarget*.

