# Supplementary material for: Blockade of Wnt/β-Catenin Pathway Aggravated Silica-Induced Lung Inflammation through Tregs Regulation on Th Immune Responses
Source: Mediators Inflamm. 2016 Mar 16;2016:6235614. doi: 10.1155/2016/6235614 (PMC4812397; doi:10.1155/2016/6235614)
Supplement: Supplementary file 1 — Figure S1: Th17 response was enhanced by blocking the Wnt/β-catenin pathway. Expression of IL-21 (A) and RORγt (B) in lung were assayed by realtime RT-PCR using the −ΔΔCt method. Values are shown as mean ± SEM (n=4-5). (∗, as compared with saline group at the same time point, p<0.05; #, as compared with silica group and silica+NC shRNA group at the same time point, p<0.05). [file 6235614.f1.pdf]

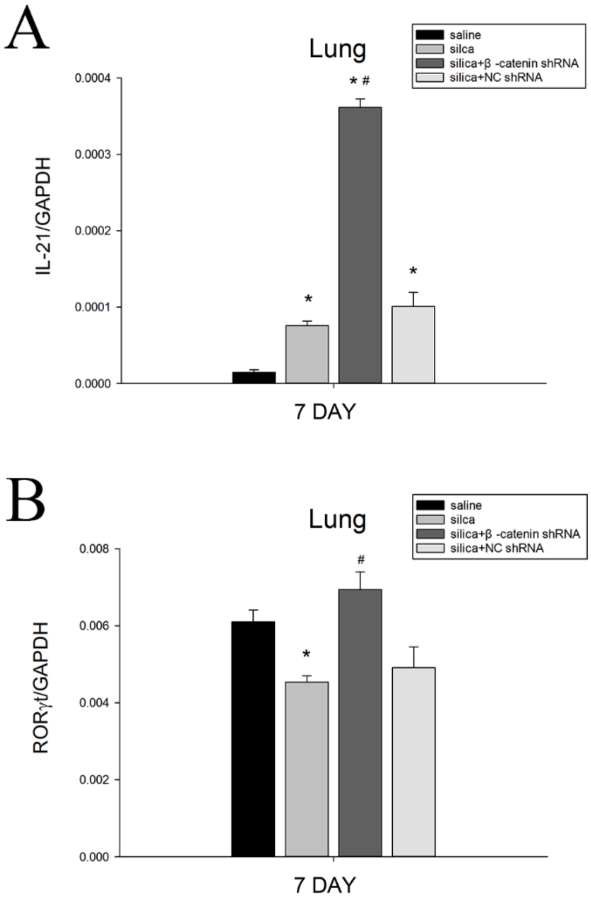

Fig. S1. Th17 response was enhanced by blocking the Wnt/β-catenin pathway. Expression of IL-21 (A) and RORγt (B) in lung were assayed by realtime RT-PCR using the -ΔΔCt method. Values are shown as mean±SEM (n=4-5). (*, as compared with saline group at the same time point, $p<0.05$; #, as compared with silica group and silica+NC shRNA group at the same time point, $p<0.05$).
